# Effects of planted pollinator habitat on pathogen prevalence and interspecific detection between bee species

**DOI:** 10.1038/s41598-022-11734-3

**Published:** 2022-05-12

**Authors:** Hannah K. Levenson, David R. Tarpy

**Affiliations:** 1 Department of Entomology & Plant Pathology, North Carolina State University, Campus Box 7613, Raleigh, NC 27695-7613 USA; 2 Biology Graduate Program—Ecology & Evolution, North Carolina State University, Campus Box 7613, Raleigh, NC 27695-7613 USA; 3 Present Address: Department of Applied Ecology, North Carolina State University, Campus Box 7617, Raleigh, NC 27695-7617 USA

**Keywords:** Community ecology, Ecological epidemiology

## Abstract

Shared resources can instigate pathogen spread due to large congregations of individuals in both natural and human modified resources. Of current concern is the addition of pollinator habitat in conservation efforts as it attracts bees of various species, potentially instigating interspecific sharing of pathogens. Common pathogens have been documented across a wide variety of pollinators with shared floral resources instigating their spread in some, but not all, cases. To evaluate the impact of augmented pollinator habitat on pathogen prevalence, we extracted RNA from samples of eight bee species across three families and screened these samples for nine pathogens using RT-qPCR. We found that some habitat characteristics influenced pathogen detection; however, we found no evidence that pathogen detection in one bee species was correlated with pathogen detection in another. In fact, pathogen detection was rare in wild bees. While gut parasites were detected in 6 out of the 8 species included in this study, viruses were only detected in honey bees. Further, virus detection in honey bees was low with a maximum 21% of samples testing positive for BQCV, for example. These findings suggest factors other than the habitat itself may be more critical in the dissemination of pathogens among bee species. However, we found high relative prevalence and copy number of gut parasites in some bee species which may be of concern, such as *Bombus pensylvanicus*. Long-term monitoring of pathogens in different bee species at augmented pollinator habitat is needed to evaluate if these patterns will change over time.

## Introduction

Shared resources can pose health risks to organisms; this is true for naturally occurring resources such as mating grounds or watering holes^1^, but also for human modified resources such as supplemental wildlife feed^2^, hunter attractants^3^, and even bird feeders^4^. These shared resources can result in dense congregations of individuals^2^ potentially causing them to act as “hotspots,” leading to pathogen build up that can then spread throughout the environment^1^. Further, the interspecific spread within these congregations can intensify if resources are scarce or limited^5^. In some cases, the resource itself can harbor pathogens^6^, increasing pathogen spread within populations^7^. However, interspecific and intraspecific pathogen spread depends on the host competency of the individual and the species for each pathogen in question^1^. Rather than acting as a hotspot, an incompetent host in a biologically diverse community at a shared resource could act to dilute the spread of a pathogen^8^.

Pollinator population declines^9^ have been repeatedly suggested to be driven by factors including agricultural intensification, nutritional stress, habitat alteration and fragmentation, and pathogens^10^, all of which can interact synergistically. Habitat loss in particular has arguably received the most attention in recent years. To combat this, augmenting habitat to support pollinators is becoming an increasingly popular conservation tool, especially in agricultural settings. While such habitat has been found to support pollinator abundance and diversity^11,12^, it is being implemented *en masse* with limited scientific evidence for best practices^13^. Evaluating the impacts of this habitat on bee populations and bee health is critical to ensure that we are not exacerbating the exact pressures that are intended to be alleviated.

Parallels can easily be drawn between human modified pollinator habitat to support bees and the shared resource examples of watering holes, supplemental wildlife feed, and bird feeders. There is a great wealth of previous literature exploring the potential for pathogen cross-over among bee species (Tables 1 and 2), particularly because similar pressures are of concern; for example, there is evidence that high abundance of common species can intensify pathogen occurance^14^. Additionally, certain flower species have been found to harbor pathogens^15,16^; however, this could be counteracted or ameliorated with habitat characteristics^17^, such as increased flower community diversity^18^. Many studies have investigated the interspecific spread of pathogens from honey bees to wild bees^19,20^, specifically. However, as differing results have been documented in differing situations (Tables 1 and 2), it begs the question: will augmented pollinator habitat act to congregate individuals leading to hotspots of pathogen spread, or will these habitats attract a diverse pollinator community leading to pathogen dilution? And what role do the habitats themselves play in pathogen spread or dilution? To evaluate how pollinator habitat influences pathogen dissemination within bee communities, we evaluated the pathogen prevalence in eight bee species from three families across 2 years. To do this, we sampled newly established pollinator habitat across North Carolina as part of the North Carolina Department of Agriculture and Consumer Services’ (NCDA&CS) mandate titled “Protecting NC Pollinators.” We investigated pathogen occurrence and prevalence within *Apis mellifera*, within *Bombus impatiens*, between *Apis mellifera* and *Bombus impatiens*, and within six other bee species that have rarely if ever been quantified in this context.

**Table 1:** A summary of previous screenings of bees for interspecific pathogen detection.

Technique	Sample Processing	Reference	Pub. Year	Location	No. of Species	*Apis*	*Bombus*	Other	Template (μl)	Max Cycle No	No. of Path. Tested	BQCV	DWV	IAPV
Infection Validation	Pool	^23^	2013	UK	1		*		1	35	1			
	Indiv	^24^	2006	DE	2		*		5	35	1		*	
		^25^	2019	DE	24	*	*	*	NR	35	1			
		^26^	2019	CH	2	*		*	NR	40	1			
		^27^	2020	N/A	2	*	*		2	35	1			
		^28^	2020	NL	2	*	*		2	40	3	*		
RT	Pool	^29^	2013	AR	1		*		5	40	7	*	*	*
		^30^	2016	BE	4		*	*	1	35	12			
		^31^	2017	CA	2	*		*	2	40	7	*	*	*
		^32^	2018	BE	8		*	*	1	35	10	*		
		^33^	2020	TX, US	15			*	NR	30	6	*	*	*
		^34^	2021	FR	30	*	*	*	NR	35	7			
Both		^35^	2011	JP	2	*			NR	35	7	*	*	*
		^36^	2013	EU	3		*		1–2	NR	9		*	
		^20^	2014	BE	6	*		*	1–3	35	16	*	*	
r-t	Indiv	^37^	2009	AR	6		*		5	30	4			
		^38^	2012	UK	17	*	*	*	1	40	4		*	
		^39^	2014	EN	7		*		5	35	6		*	
		^40^	2015	N/A	2	*	*		1–2	35	5		*	
		^41^	2018	US	28		*		1	40	3			
		^14^	2020	NY, US	9	*	*	*	1	40	5			
RT		^42^	2012	PA, US	15		*	*	NR	38	5	*	*	*
		^43^	2011	UT, US	1		*		NR	40	1	*		
		^44^	2013	PA, US	30		*	*	5	38	5	*	*	*
		^45^	2014	G.B	2	*	*		NR	NR	2		*	
		^46^	2015	CO	1		*		1	35	10	*	*	
		^47^^1^	2017	DE	33	*	*	*	NR	40	6	*		
		^48^	2019	NY, US	2	*		*	NR	NR	3	*	*	
		^49^	2020	NZ	24	*	*	*	1	35	5	*	*	
		^50^	2020	NE, US	4	*	*		2.5	40	4	*	*	*
		^51^	2021	MI, US	4	*	*	*	2–2.5	37	3			
Both		^52^	2018	PL	4		*		1–3	35	6		*	
		^53^	2019	AR	3		*		5	35	10	*	*	*
RT	Both	^54^	2019	IT	1			*	5	50	7	*		*

A two-letter code is used for each country, with a two-letter state code also included for US projects. The total number of bee species tested is shown, followed by if certain common species were included. Similarly, the total number of pathogens tested is shown, followed by if certain commonly tested for pathogens were included. This table shows papers that tested for infection validation or used ‘traditional’ PCR techniques (real time PCR [r-t], reverse transcription [RT] PCR, or Both r-t and RT) only. When a piece of information was not reported, it is shown as NR in the table. Max cycle number refers to the cycle number used during PCR analysis.

^1^This paper used capillary electrophoresis to score their RT-PCR.

**Table 2:** Similar to Table 1, this table summarizes previous screenings of bees for interspecific pathogen detection.

Standard Used	Sample Processing	Reference	Pub. Year	Location	No. of Species	*Apis*	*Bombus*	Other	Template (μl)	Max Cycle No	No. of Path. Tested	BQCV	DWV	IAPV
No	Pool	^55^	2011	UT, US	1		*		NR	30	1		*	
		^56^	2015	MX	1		*		1	40	10		*	*
		^57^	2021	PA, US	3	*	*	*	2	35	5		*	*
Yes		^58^	2016	IA, US	5	*		*	NR	40	5		*	*
		^59^	2018	IT	1			*	5	50	1		*	
		^19^	2019	VT, US	3	*	*		NR	40	3	*	*	*
No	Indiv	^60^^1^	2020	PE; BO	3		*		NR	NR	5			
Yes		^61^	2015	G.B	2	*	*		NR	40	6	*	*	
		^62^^2^	2018	UK	5	*		*	2–2.5	45	5	*		
		^63^	2021	IA, US	3	*	*		NR	45	3			
	Both	This paper	2022	NC, US	8	*	*	*	1	40	9	*	*	*

This table shows papers that used quantified PCR techniques only and notes if each paper included a standard curve during analysis.

^1^This paper used qPCR.

^2^This paper used RT-PCR for some targets and RT-qPCR for others.

## Materials and methods

### Sample collection

Samples were collected at established pollinator habitat at 12 sites across North Carolina (Supplemental Tables 1 and 2) in 2017 and 2018. Collection events occurred once a month for 4 months during peak bloom at each plot, for a total of four sampling events per locations per year (hereafter referred to as Spring, Early Summer, Late Summer, and Fall), utilizing hand nets for 30 ± 10 min along haphazard transects^21^. All samples were collected between April and November (Supplemental Tables 1 and 2). Focus was placed on the most commonly occurring species to ensure sufficient replication. Each individual bee collected was placed into a separate 1.7 ml microcentrifuge tube and transported back to the lab on dry ice where they were then stored at − 80 °C until further processing. At each station during each sampling event, the flower cover and flower diversity within the plot was documented and categorized into low, medium, or high. Flower cover was categorized based on what percentage of the sampling plot was in bloom at the time the samples were collected, with 0–30% bloom corresponding to low, 31–50% to medium, and 51% or more to high. Flower diversity was categorized based on how many different plant species were in bloom at the time the samples were collected, with low corresponding to 80–100% of the plot in bloom with one flower species, medium to 60–79%, and high to 59% or less (see Levenson and Tarpy^22^ for more details).

### Pathogen screening

Eight different bee species (Apidae: *Apis mellifera, Bombus impatiens, Bombus pensylvanicus, Svastra obliqua, Xylocopa virginica, Xylocopa micans*; Halictidae: *Halictus poeyi/ligatus*; and Megachilidae: *Megachile xylocopoides*; Table 3) were screened for 9 different pathogens (acute bee paralysis virus [ABPV], black queen cell virus [BQCV], chronic bee paralysis virus [CBPV], deformed wing virus A [DWVa], deformed wing virus B [DWVb], Israeli acute paralysis virus [IAPV], Lake Sinai virus [LSV], *Trypanosome* universal primer [*Trypanosome* spp.], and *Vairimorpha* primer [as a *Nosema* universal primer was used during screening, results from this target will henceforth be referred to as *Nosema* spp. for simplicity]; as well as two reference genes (*actin* and *apocrita 28 s* [*apo28s*]; Supplemental Table 3). The *Trypanosome* universal primer was designed to amplify *Chrithidia mellificae, Chrithidia bombi,* and *Lotmaria* passim. The *Nosema* universal primer was designed to amplify *Nosema apis* and *Nosema ceranae*. All primer working stocks were diluted to 5 mmol. North Carolina is on the border of the range for *H. poeyi* and *H. ligatus*; because these two species are cryptic species and morphologically identical^64^ samples of this species complex are referred to as *H. poeyi/ligatus.*

**Table 3:** A summary of the number of individuals and pools screened for each bee species during pathogen analysis.

		Sample Number		Number of Samples with Positive Detections									Number of Pathogens
Species	Sample Status	Screened	Total Individuals	ABPV	BQCV	CBPV	DWVa	DWVb	IAPV	LSV	*Try.* spp.	*Nos.* spp.	Total Detected
*Apis mellifera*	Individual	189	189	1	40	–	32	8	1	27	26	22	8
*Bombus impatiens*	Individual	201	201	–	–	–	–	–	–	–	68	1	2
*Bombus pensylvanicus*	Pooled	19	31	–	–	–	–	–	–	–	6	5	2
*Halictus poeyi/ligatus*	Pooled	58	260	–	–	–	–	–	–	–	6	–	1
*Megachile xylocopoides*	Pooled	2	3	–	–	–	–	–	–	–	–	–	0
*Svastra obliqua*	Pooled	12	28	–	–	–	–	–	–	–	1	1	2
*Xylocopa micans*	Individual	1	1	–	–	–	–	–	–	–	1	–	1
*Xylocopa virginica*	Individual	20	20	–	–	–	–	–	–	–	–	–	0
	Totals	502	733	1	40	0	32	8	1	27	108	29	

The number of samples screened, total number of samples included, number of positive detections for each pathogen, and total number of pathogens detected are shown.

Seven of the pathogens examined in this study are viruses and were selected because they are some of the most commonly occurring honey bee pathogens that have been shown to negatively affect honey bee health^65^. Although little is known about the true impact of most of these pathogens on native bee health and longevity^14^, transmission is likely given that bee species in the same area of a study have the same virus profiles^44,61^. The remaining pathogens are gut parasites; these pathogens were selected because they are commonly detected, known to negatively impact bee survival^66^ and so are economically important, interspecific transmission has been previously documented^23^, and infection of gut parasites has been linked to population losses in some cases^67^.

### Sample preparation: individual bee samples

Samples of *A. mellifera* and *B. impatiens* were processed as individuals as the sample sizes of these species were the highest in our study. Samples of *X. virginica* and *X. micans* were also processed as individuals due to their large body size. When processing these individual samples, we removed each specimen from cold storage and kept it on dry ice until crushed, following an adapted protocol from Leite et al. 2012^68^ to ensure successful pulverization and the highest quality RNA due to sample brittleness. We used two Zirconium beads (3.0 mm) for *A. mellifera* and *B. impatiens* and three Zirconium beads for *X. virginica* and *X. micans*, placing each tube into the Ivoclar Silamat S6 in order to crush the sample. Once completely pulverized, we extracted RNA using the TRIzol^®^ Reagent^69^ and the Zymo Direct-zol™ RNA Miniprep Kit, following the Directzol protocol. After extraction, we assessed RNA quantity and quality using the Thermo Scientific NanoDrop ND-1000 Spectrophotometer and diluted to 200 ng of RNA per microliter. All RNA was again stored at − 80 °C until further analysis.

### Sample preparation: pooled bee samples

Due to sample size and low pathogen detection (discussed below), we tested *B. pensylvanicus, H. poeyi/ligatus, S. obliqua,* and *M. xylocopoides* in pools of up to five individuals (depending on how many were collected during each sampling event) using whole bodies (summarized in Table 3). To process pooled samples, we took up to five bees per sampling event per location out of ultracold storage and immediately placed them into a sanitized ceramic mortar. Sufficient liquid nitrogen was immediately added to cover all bee material and allowed to sublimate to ensure that the samples were brittle. We then immediately and quickly pulverized samples using a pestle. Once completely crushed, we filled a new 1.7 µl tube approximately halfway with the powdered materials so as to leave enough space for the TRIzol^®^ Reagent. As individuals of *H. poeyi/ligatus* are small, we combined these pooled samples into one new 1.7 tube and crushed them using the Zirconium bead protocol described above for individual samples. RNA extraction of pooled samples followed the same protocol as described above.

### Sample testing

To determine the concentrations of pathogen infections in the samples, we used a two-step reverse transcriptase quantitative PCR (RT-qPCR) analysis. In step one, we synthesized cDNA with a 10-µl reaction volume using the BioBasic High Reverse Transcriptase Kit (Biobasic, Marhkam, Canada). Each well received 1 µl of extracted RNA (at a concentration of 200 ng per microliter), 2 µl of master mix, and 7 µl of lab grade water; after, we diluted the cDNA with 50 µl of water. The resulting cDNA contained a relative concentration of 3.3 ng of RNA per microliter assuming full conversion to cDNA. In step two, we performed real time PCR in triplicate on 384-well plates using Life Technologies PowerUp SYBER Green chemistry with a Quant Studio 6 Flex machine. We used 3.2 µl reaction volumes that included 1 µl of template cDNA, 1.5 µl of SYBER, 0.15 µl of primer (forward and reverse combined), and 0.55 µl of water. We included standards for absolute quantification in each plate, which involved a serial dilution of known quantities of a custom synthesized plasmid containing the targets, with one negative control containing only water also included. Even though under this protocol each plate completed 40 cycles during the PCR stage, we only included positive results that were within the range of the quantified standards. If a sample contained a positive result at a cycle number higher than the positive standards, it was not considered to be biologically relevant. Thus, the cycle number cutoff ranged from 28 to 32 cycles, depending on the target and the specific target’s standard’s results. We performed analyses using the included Quant Studio software and then normalized results to the reference gene levels using GeNorm^70^.

In 2018, we collected a subset of the flowers on which the bees were foraging and conducted pathogen screening in order to determine if they contained similarly detectable levels of the pathogens. Five flower heads per sampling event were removed, placed in individual bags, and transported back on dry ice as was done with the bee samples. We screened these flowers for pathogens as detailed above; however, no pathogens were detected (data not included) and as such we did not analyze these data.

### Statistical analysis

Since there is an overdispersion of zeros in our dataset, we used a Zero Inflated Negative Binomial model (ZINB)^71^ with a logit link. Detection levels of pathogens were analyzed in two ways; copy number (standardized to the reference gene), and relative intensity (categorized into non-detect (ND) if zero, and low, medium, or high based on the bottom two, third, and fourth quartiles of the natural log transformed copy numbers of each pathogen, respectively; Supplemental Table 4). Relative intensity was calculated within each target, not across targets. To explore the potential pathogen dissemination between *A. mellifera* and *B. impatiens*, we included season, flower cover, and flower diversity as independent variables and pathogen copy number as dependent variables in a ZINB model. We then used an ANOVA in base R to compare *A. mellifera* presence and relative intensity to *B. impatiens* presence and relative intensity*.*

When constructing our ZINB models, year was not found to significantly impact pathogen detection in *A. mellifera* (all *p* > 0,34), except for BQCV detections (*p* < 0.05). As such, year was only included as a random effect in models when analyzing BQCV detection in *A. mellifera*. Additionally, sampling location was not found to significantly impact pathogen detection in *A. mellifera* (all *p* > 0.16), except for *Nosema* spp. detections (*p* < 0.0001). However, in order to maintain statistical power, it was not included in any of our models. Both year (*p* < 0.0001) and station (*p* < 0.005) were found to significantly impact *Trypanosome* spp. detections in *B. impatiens*, however through an AIC based approach for best model selection these variables were not included in our final model.

Due to low sample size and low pathogen presence, we were not able to conduct further analyses on the pathogen results from the remaining six bee species; however, these findings are summarized descriptively below. All analyses were conducted in RStudio (version 3.6.2) using base R^72^, the *pscl*^73^ package, and the *boot*^74^ packages.

## Results

**Figure 1:**
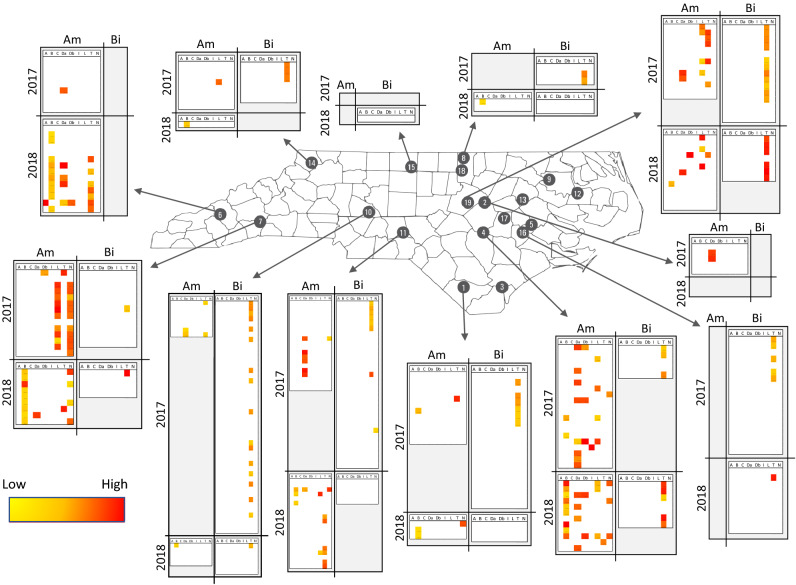
Visual comparison of viral matrices. Each block shows samples from a particular location with *A. mellifera* (Am) [on the left-hand matrix of each block] and *B. impatiens* (Bi) [on the right-hand matrix]. Samples from 2017 are displayed at the top of each block and 2018 on the bottom. Each row represents an individual sample, and each column represents a different target listed in alphabetical order (A = ABPV; B = BQCV; C = CBPV; Da = DWVa; Db = DWVb; I = IAPV; L = LSV; T = *Try.* spp.; and N = *Nos.* spp.). Relative intensity is represented with a color gradient from low (bright yellow) to high (bright red). This figure was created using Adobe Illustrator, Microsoft Excel, and Microsoft Word. Thank you to Kirsten Benson for creating the base map.

We originally collected and screened 616 bee samples; however, we removed 114 samples from analysis as the amplification levels of one or both of the reference genes were at an unacceptably low level. As such, we included a total of 502 samples in our analysis—411 individually processed samples and 91 pooled samples (Fig. 1 and Table 3).

A visual representation of the results for *Apis mellifera* and *Bombus impatiens* are displayed in Fig. 1. Each block displays the samples screened at each sampling location. Within each block, results are displayed for both *A. mellifera* (the left-hand matrix of each block) and *B. impatiens* (the right-hand matrix of each block) as well as for each year (2017 results are shown on the top half of each block and 2018 results on the bottom). Each row within a viral matrix represents an individual sample tested. Each column represents the target that sample was screened against listed in alphabetical order (A = ABPV; B = BQCV; C = CBPV; Da = DWVa; Db = DWVb; I = IAPV; L = LSV; T = *Try.* spp.; and N = *Nos.* spp.). Thus, each cell displays whether a target was detected in a specific sample. While we calculated pathogen presence discretely with quartiles (Supplemental Table 4), we display those results here on a continuous gradient where no detection of a target is represented in white, low detection of a target (corresponding to Q1 and Q2) is represented in yellow, medium detection of a target (Q3) is represented in shades of orange, and high detection of a target (Q4) is represented in red. As each target may be detected at different efficiencies during testing, these quartiles are calculated within each target, not across targets.

**Figure 2:**
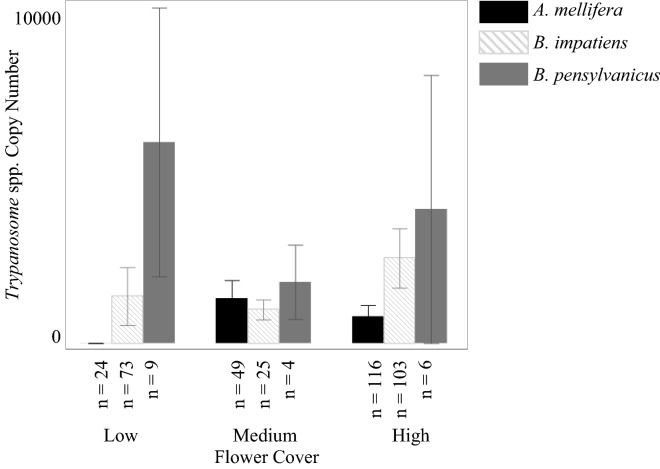
Copy number per 1 µl of template (approximately 3.3 ng of RNA) of *Trypanosome* spp. for *A. mellifera, B. impatiens,* and *B. pensylvanicus* across the different levels of flower cover.

**Figure 3:**
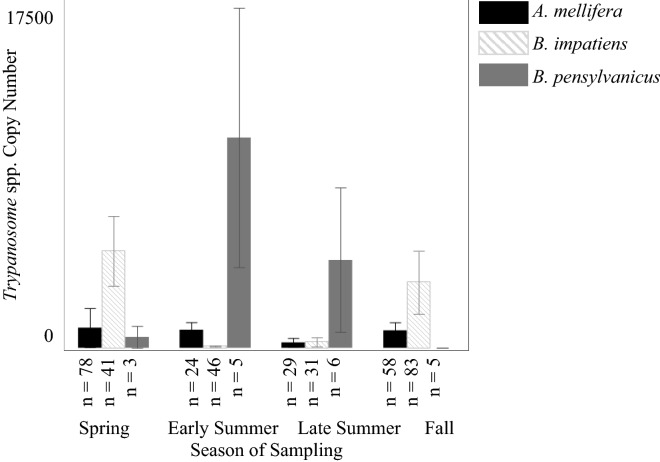
Copy number per 1 µl of template (approximately 3.3 ng of RNA) of *Trypanosome* spp. for *A. mellifera, B. impatiens,* and *B. pensylvanicus* across the sampling season.

*Apis mellifera* was the only bee species in which we detected any of the viruses in our study. The most commonly detected pathogen in *A. mellifera* was BQCV (40 individuals), followed by DWVa (32), LSV (27), *Trypanosome* spp. (26), *Nosema* spp. (22), ABPV (1), IAPV (1), and finally CBPV with no detections (Table 3). Further, many individuals were found to be simultaneously infected with multiple pathogens, with two individuals infected with four pathogens (Fig. 3). We found that LSV had the highest copy number overall, but that BQCV (29.0% of positive detections) and *Trypanosome* spp. (52.9% of positive detections) more often fell into the high category of relative intensity. Due to low, or no, positive detections, we were unable to analyze ABPV, CBPV, DWVb, and IAPV results for *A. mellifera*. From the pathogens we were able to analyze, we found that BQCV copy number was significantly highest in the spring (logθ = − 1.34; DF = 11; *p* < 0.0001, SE ± 1.10), and was lowest at medium flower diversity (*p* < 0.005, SE ± 2.04). LSV did not significantly change across the sampling season or flower diversity (all *p* values > 0.18), but we detected the highest copy numbers in low flower cover (logθ = − 1.07; DF = 17; *p* < 0.0001, SE ± 1.51). Conversely, we detected the highest *Trypanosome* spp. copy number at high flower cover (logθ = − 0.25; DF = 17; *p* < 0.0005, SE ± 1.20; Fig. 2) and when flower diversity was low (*p* < 0.0001, SE ± 1.27). Additionally, copy number of *Trypanosome* spp. was highest in late summer (*p* < 0.01, SE ± 1.71; Fig. 3). To analyze the *Nosema* spp. results, flower diversity was removed from the model. We found that copy number detection level of *Nosema* spp. was highest in fall (logθ = − 0.44; DF = 13; *p* < 0.001, SE ± 1.63) and spring (*p* < 0.0001, SE ± 1.33) and was not significantly impacted by flower cover (*p* = 0.64). DWVa was not significantly influenced by any of the variables in our model (all *p* values > 0.06).

We only analyzed *Trypanosome* spp. copy number within *B. impatiens* as no viruses were detected in any of our *B. impatiens* samples and only one individual was detected with *Nosema* spp. Copy numbers within *B. impatiens* (68 individuals) were higher than copy numbers in *A. mellifera*. We found that *Trypanosome* spp. copy number was significantly lowest in the fall (logθ = − 0.85; DF = 17; *p* < 0.005, SE ± 0.91; Fig. 3) and significantly highest with medium flower diversity (*p* < 0.05, SE ± 0.88) and low (*p* < 0.01, SE ± 0.70; Fig. 2) flower diversity. *Trypanosome* spp. copy number was not significantly influenced by flower cover (all *p* values > 0.24).

When exploring potential pathogen dissemination between *A. mellifera* and *B. impatiens* we focused on *Trypanosome* spp. detections, as this was only pathogen detected in both species with high sample numbers. We did not find any evidence that pathogen detection of one species was correlated with the pathogen detection of the other. Presence of a positive *Trypanosome* spp. detection in *A. mellifera* had no correlation with any relative intensity category in *B. impatiens* (all *p* values > 0.15). Similarly, the relative intensity of *Trypanosome* spp. in *A. mellifera* had no correlation to the presence or relative intensity of *Trypanosome* spp. in *B. impatiens* (all *p* values > 0.18).

While we did not find any positive detections of viruses in the other bee species tested in this study, we did find gut pathogens. *Trypanosome* spp. were detected in *B. pensylvanicus* (6 pools)*, H. poeyi/ligatus* (6)*, S. obliqua* (1)*,* and *X. micans* (1 individual; Table 3 and Figs. 2 and 3). *Nosema* spp. was also detected in *B. pensylvanicus* (5 pools) and *S. obliqua* (1). Within these gut pathogen results, *B. pensylvanicus* had the highest copy number detection level for *Nosema* spp., by an entire order of magnitude, followed by *S. obliqua* and then *A. mellifera*. *Bombus pensylvanicus* had the highest copy number detection level of *Trypanosome* spp. again followed by *S. obliqua,*and then *B. impatiens*.

## Discussion

*Apis mellifera* was the only pollinator species in which we detected any viruses. However, we detected gut pathogens across most of the bee species tested. Some pathogen copy numbers—such as BQCV and *Nosema* spp. in *A. mellifera*, and *Trypanosome* spp. in both *A. mellifera* and *B. impatiens*—significantly changed across the sampling season, a finding that is similar to previous literature^17^. While other pathogen copy numbers—such as LSV in *A. mellifera* and *Trypanosome* spp. in both *A. mellifera* and *B. impatiens*—were significantly influenced by flower cover; however, this occurred in opposite directions where LSV was highest at low flower cover and *Trypanosome* spp. were highest at high flower cover. Similar to previous literature^75^, *Trypanosome* spp. detection levels were highest in low flower diversity. While *Trypanosome* spp. detection patterns were similar in *A*. *mellifera* and *B. impatiens*, we found no evidence of correlations between these two species. These results suggest that the habitat is not acting as a pathogen hotspot but rather some other mechanism may be more critical in pathogen dissemination within bee communities. One explanation could be that even though shared floral resources have been documented as a source of spread for some pathogens^40^, the occurrence may actually be rather rare^16^ and its success depends on the bee^15^ and flower species in question^16^. It has also been suggested that non-host bees can reduce infection levels through the dilution effect^51^. It is possible that as time progresses and bees continue to utilize these habitats, the pathogens pressures will intensify intraspecifically. Further long-term testing will be necessary to evaluate this possibility.

Gut parasites are currently considered a serious threat to several bee species, especially bumble bees^67^; of particular concern in North America is the American Bumble Bee (*B. pensylvanicus*). In our study, *B. pensylvanicus* had the greatest positive detections of gut parasites out of all the bee species tested, supporting the hypothesis that gut parasites pose a threat to their populations. At the time of writing this paper—but after the period when samples were collected and analyzed—the United States Fish and Wildlife Service (FWS) has announced a 90-day findings petition for *B. pensylvanicus* populations in order to inform decisions surrounding its population status, and status reviews are underway in state FWS offices. Currently in North Carolina, *B. pensylvanicus* is listed at “W3: Rare but Questionable Documentation”^76^ and “Vulnerable/Apparently Secure”^76^, meaning more documentation is needed on this species before making any regulatory decision. Information from this study will be important in making future conservation decisions surrounding this and other species, and data from this study has already been shared with the NCFWS to do so. As gut pathogens are considered a threat to this species’ population^77^, monitoring should be continued in future work. However, one consideration is noting the species of gut pathogens being detected. While all samples in this study were screened for *N. apis* and *ceranae*, preliminary results showed that some samples tested positive for the *Nosema* spp. primer but did not test positive for *N. ceranae*; however, as the results were inconclusive, the data are not included here. This suggests native bee populations are facing their own gut parasites, especially *Nosema* species^78^, that are not as commonly tested for. This has already been documented in previous literature^32^, especially in bumble bees with regards to the health consequences of *Nosema bombi* infections^79^. It is important to note, though, that detecting a pathogen neither equates to infection nor demonstrates specific health impacts of the pathogen^28^. For example, it has been suggested that the presence of *N. ceranae* in *B. terrestris* may be due to ingested spores passing through the gut rather than true infection^27^. Future research should prioritize evaluating the true infectivity and health impacts of these pathogens on a variety of bee species, taking into consideration the use of species specific pathogen primers.

Many studies have previously found the presence of what are traditionally called ‘honey bee’ viruses in various native bee species, something this study does not confirm. Given that several other recently published papers have also documented fewer detections than previous research^34,57,63^, the unexpected results require speculation as to why. Unlike most other studies, we collected honey bee samples as individual foragers rather than groups from nest entrances or even inside managed beehives. This could have resulted in lower infection levels in our samples (e.g., heavily infected bees may not live long enough or be sufficiently healthy to forage) resulting in reduced pathogen detection and spread. Another factor that plays a key role is viral sequence variation. Previous research has documented different variants circulating in different regions of the world^18^. The presence of different viral variants could reduce interspecific spread between bee species and also impact detectability of such variants. Alternatively, floral diversity has been documented as an important factor for pathogen sharing and infection levels^18,75^. Thus, plant diversity could potentially be used as a tool to intentionally limit pathogen sharing between honey bees and native bees at these augmented habitats. This is something that should be investigated further in future research and taken into consideration when establishing new pollinator habitat.

Another factor to consider when comparing the results from this study to previously published work is the techniques and methods used to screen for pathogens. Nearly every aspect of sample processing could influence the detection of pathogens including the brand of chemical used, primer efficiencies, the amount of starting template, specific techniques used, and even machine functioning. In a study on mouse behavior it was found that lab environment alone caused significant differences in results, even when standardizing protocols^80,81^. As noted in Tables 1 and 2 of this paper, previous research evaluating co-occurrence of pathogens among honey bees and other species has varied in technique used, starting template amount, and sample processing—all of which could bias results. When coupled with other considerations such as spurious PCR amplification (as is known to occur at 30 cycles and above^82^) and the influence of pathogen infection itself on detection ability within a sample^83^, comparing across studies should be done with extreme caution so as to avoid potentially misleading comparisons.

As planted habitat for pollinators will likely continue to be used as a tool in pollinator conservation, we should take care to establish this habitat with plant species that provide floral resources while limiting pathogen transmission. We should also prioritize conducting long-term monitoring of the bees within these habitats to ensure it continues to protect pollinator populations and their health over time.

## Supplementary Information

Supplementary Information 1.

Supplementary Information 2.

Supplementary Information 3.
