# Supplementary material for: Effects of planted pollinator habitat on pathogen prevalence and interspecific detection between bee species
Source: Sci Rep. 2022 May 12;12:7806. doi: 10.1038/s41598-022-11734-3 (PMC9098541; doi:10.1038/s41598-022-11734-3)
Supplement: Supplementary file 3 — Supplementary Information 3. [file 41598_2022_11734_MOESM3_ESM.docx]

**Effects of planted pollinator habitat on pathogen prevalence and interspecific detection between bee species**

Hannah K. Levenson (ORCID: 0000-0002-1667-0127) ^1,2*^ and David R. Tarpy (ORCHID: 0000-0001-8601-6094) ^1,2,3^

^1^Department of Entomology & Plant Pathology

^2^Biology Graduate Program—Ecology & Evolution

Campus Box 7613

North Carolina State University

Raleigh NC 27695-7613

^3^Current address: Department of Applied Ecology

Campus Box 7617

North Carolina State University

Raleigh NC 27695-7617

^*^Author for correspondence:

TEL: 919-515-3967

FAX: 919-515-7746

EMAIL: [hklevens@ncsu.edu](mailto:hklevens@ncsu.edu)

Keywords: bee, pathogen, bee health, shared floral resources, pollinator habitat

**Supplemental Table 3.** Information on the tested pathogens. This table outlines each target tested, showing the full name, abbreviated name, forward sequence, reverse sequence, and citation where the primer sequences are from. The parasite species each universal primer was designed to amplify are listed below, however it is possible that other species could be amplified by these primers. Primers citation: Milone, J.P., Tarpy, D.R. Effects of developmental exposure to pesticides in wax and pollen on honey bee (*Apis mellifera*) queen reproductive phenotypes. Sci. Rep**.** 2021; 11.

| Target | Target Name | Sequence (5’ – 3’) | Amplicon Size | Efficiencies |
| --- | --- | --- | --- | --- |
| **ABPV** | acute bee paralysis virus | F: TCCCAAGATTGGAATAAGACAGTTAG | 108 bp | 87.95% |
|  |  | R: TTCCATAATGCAAACATTCAAAGATCC |  |  |
| **BQCV** | black queen cell virus | F: CGAAGCGTTTTCCGTGG | 183 bp | 85.49% |
|  |  | R: GCTGTCGAGAGTCAGAGTT |  |  |
| **CBPV** | chronic bee paralysis virus | F: ACTGCTGCCCTCGATAG | 77 bp | 93.30% |
|  |  | R: TGTGTTGAGGCAGGTTGG |  |  |
| **DWVa** | deformed wing virus – strain A | F: GTCTTGTGGATGAAGGTTATATAACTGG | 169 bp | 89.28% |
|  |  | R: TCCGTAGAAAGCCGAGTTG |  |  |
| **DWVb** | deformed wing virus – strain B | F: ACCAACGCGTGTCGTTCCTG | 109 bp | 93.96% |
|  |  | R: ACAAGTGGTTGGTCCCGTCG |  |  |
| **IAPV** | Israeli acute paralysis virus | F: GCTAATACCAAGACACCAATCACGGACC | 59 bp | 91.95% |
|  |  | R: TCTCGACCCTGAGCATCTGTG |  |  |
| **LSV** | Lake Sinai virus – 15 strains | F: TSATCCMAAGAGAACCACT | 70 bp | 69.24% |
|  |  | R: CATGAAGAAATGAGGKCCGC |  |  |
| **Try. spp.** | *Trypanosome* universal – *Crithidia mellificae, Crithidia bombi, Lotmaria passim* | F: GAGTGTGGCAGGACTACCC | 169 bp | 82.47% |
|  |  | R: TGCACCAACCACGAAATGA |  |  |
| **Nosema spp.** | *Nosema* universal – *Nosema apis, Nosema ceranae* | F: AGCAGCCGCGGTAATACTTGTTC | 144 bp | 87.94% |
|  |  | R: GTTCGTCCAGTCAGGGTCGT |  |  |
| **actin** | *Apis mellifera actin* reference gene | F: TTGGCTGGCCGTGATTTGAC | 236 bp | 85.83% |
|  |  | R: GGACAACGGAATCTTTCGTTACCAAT |  |  |
| **apo28s** | *apocrita 28 s* reference gene | F: TGGTTCCCTCCGAAGTTTCCCTCAG | 158 bp | 86.12% |
|  |  | R: GCAAGCCAGAGATCTCACCCATTTA |  |  |

**Supplemental Table 4.** Details on the quartile ranges for each target with the upper cut off reported. These quartiles correspond to low detection (Q1 and Q2), medium detection (Q3) and high detection (Q4). Quartiles are calculated by the natural log transformed copy number but are reported in untransformed copy numbers here. Approximately 3.3 ng per microliter of RNA was used during RT-qPCR to calculate these copy numbers. These quartiles are calculated within each target, not across targets.

| Target | Low Detection Copy Number Cut Off  (Q1, Q2) | Medium Detection Copy Number Cut Off  (Q3) | High Detection Copy Number Cut Off  (Q4) |
| --- | --- | --- | --- |
| ABPV | N/A | N/A | 1054 |
| BQCV | 128 | 598 | 128419 |
| CBPV | N/A | N/A | N/A |
| DWVa | 8459 | 18012 | 92532 |
| DWVb | 8209 | 18588 | 654331 |
| IAPV | N/A | N/A | 135 |
| LSV | 17227 | 126476 | 958896 |
| Try. spp. | 1058 | 5257 | 57364 |
| Nosema spp. | 82516 | 160874 | 3156420 |
